# P-649. Unusual Increase in *Bordetella parapertussis* Cases Detected in Pediatric Reference Centers in Mexico and Colombia During 2023: Is this Trend Emerging Elsewhere?

**DOI:** 10.1093/ofid/ofae631.846

**Published:** 2025-01-29

**Authors:** Ivan Felipe Gutiérrez Tobar, Ana C Carbajal-César, Juan Pablo Londono-Ruiz, Viviana Antonio-González, Daniel A Salinas-Morales, Carolina Robledo-Vega, Nicolás Cuéllar, Gloria M Gutiérrez-Reyes, Eduardo Arias de la Garza, Leonor Patricia Saltigeral Simental, Dámaris Manzano-Arredonda, Virginia Díaz-Jiménez

**Affiliations:** Clinica Infantil Colsubsidio, Clínica Infantil Santa María del Lago, Bogotá, Distrito Capital de Bogota, Colombia; Instituto Nacional de Pediatría, Ciudad de México, Distrito Federal, Mexico; Hospital universitario Mayor Mederi, Bogota, Distrito Capital de Bogota, Colombia; Instituto Nacional de Pediatría, Ciudad de México, Distrito Federal, Mexico; Star Médica Hospital Infantil Privado, México City, Distrito Federal, Mexico; Star Médica Hospital Infantil Privado, México City, Distrito Federal, Mexico; Facultad de Medicina, Pediatria, Pontificia Universidad Javeriana, Bogotá, Distrito Capital de Bogota, Colombia; Star Médica Hospital Infantil Privado, México City, Distrito Federal, Mexico; Instituto Nacional de Pediatría, Ciudad de México, Distrito Federal, Mexico; Instituto Nacional de Pediatría, Ciudad de México, Distrito Federal, Mexico; Instituto Nacional de Pediatría, Ciudad de México, Distrito Federal, Mexico; Instituto Nacional de Pediatría, Ciudad de México, Distrito Federal, Mexico

## Abstract

**Background:**

*Bordetella parapertussis* infections clinically present with symptoms similar to *Bordetella pertussis*, albeit less severe and less frequent. We describe a multinational experience of an increase in cases from three pediatric reference centers in Mexico and Colombia

Graph 1

**Figure:**
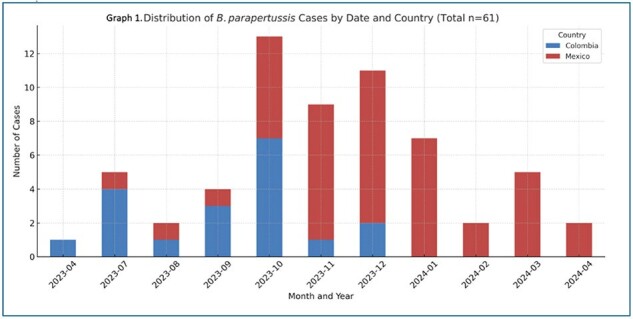

Distribution of B. parapertussis cases by date and country

**Methods:**

Observational, retrospective study conducted at 3 pediatric reference centers, 2 in Mexico and 1 in Colombia. We included patients positive for *Bordetella parapertussis* via multiplex PCR respiratory assays from January 2023 to April 2024. Analysis focused on clinical characteristics and outcomes. Descriptive statistics were utilized and statistical comparisons were performed with the Mann-Whitney U test and Fisher's exact or chi-squared tests, significance was set at p < 0.05

Graph 2

**Figure:**
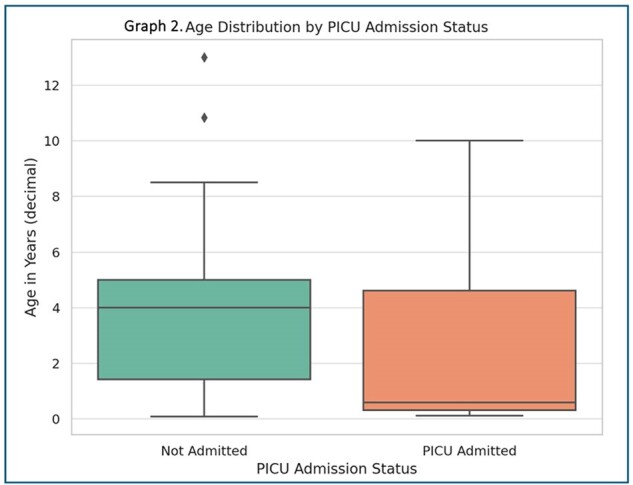

Age distribution by PICU admission status

**Results:**

The study involved 61 patients, 42 from Mexico and 19 from Colombia, with initial cases originating in Colombia (Graph 1). Median age was 3 years (1 month to 13 years), with no significant age differences between countries. Males accounted for 59% of cases. Approximately 70.49% of children were fully vaccinated, and 26.23% had contact with individuals displaying respiratory symptoms. Common symptoms included cough (90.16%), rhinorrhea(60.66%), respiratory distress (59.02%), fever (59.02%), and paroxysms (22.95%). Coinfections were prevalent in 85.25% of cases, mainly rhinovirus (45.9%) and RSV (18.0%). Laboratory findings showed median leukocyte count of 9,450 (IQR: 7,250-14,922.5).Chest X-rays indicated interstitial patterns in 58.33% and consolidation in 8.33% of cases. The PICU admitted 24.59% of patients, with 6.56% requiring intubation. A significant age difference was observed between PICU admissions (median 0.58 years) and non-admissions (median 4 years), p = 0.037 (Graph 2). No fatalities occurred.

**Conclusion:**

The increase in cases occurred in two countries, initially emerging and then subsiding in Colombia. Most patients had viral co-detection, mainly with Rhinovirus. Despite typically mild descriptions in the literature, around a quarter required ICU admission, particularly among younger patients, suggesting increased vulnerability in this group. Continuous monitoring of evolving circulation patterns is vital locally and regionally, as similar phenomena may be happening elsewhere.

**Disclosures:**

**All Authors**: No reported disclosures

